# Supplementary figures and images for: Nanopore sequencing reveals full‐length Tropomyosin 1 isoforms and their regulation by RNA‐binding proteins during rat heart development
Source: J Cell Mol Med. 2021 Jul 24;25(17):8352–62. doi: 10.1111/jcmm.16795 (PMC8419188; doi:10.1111/jcmm.16795)

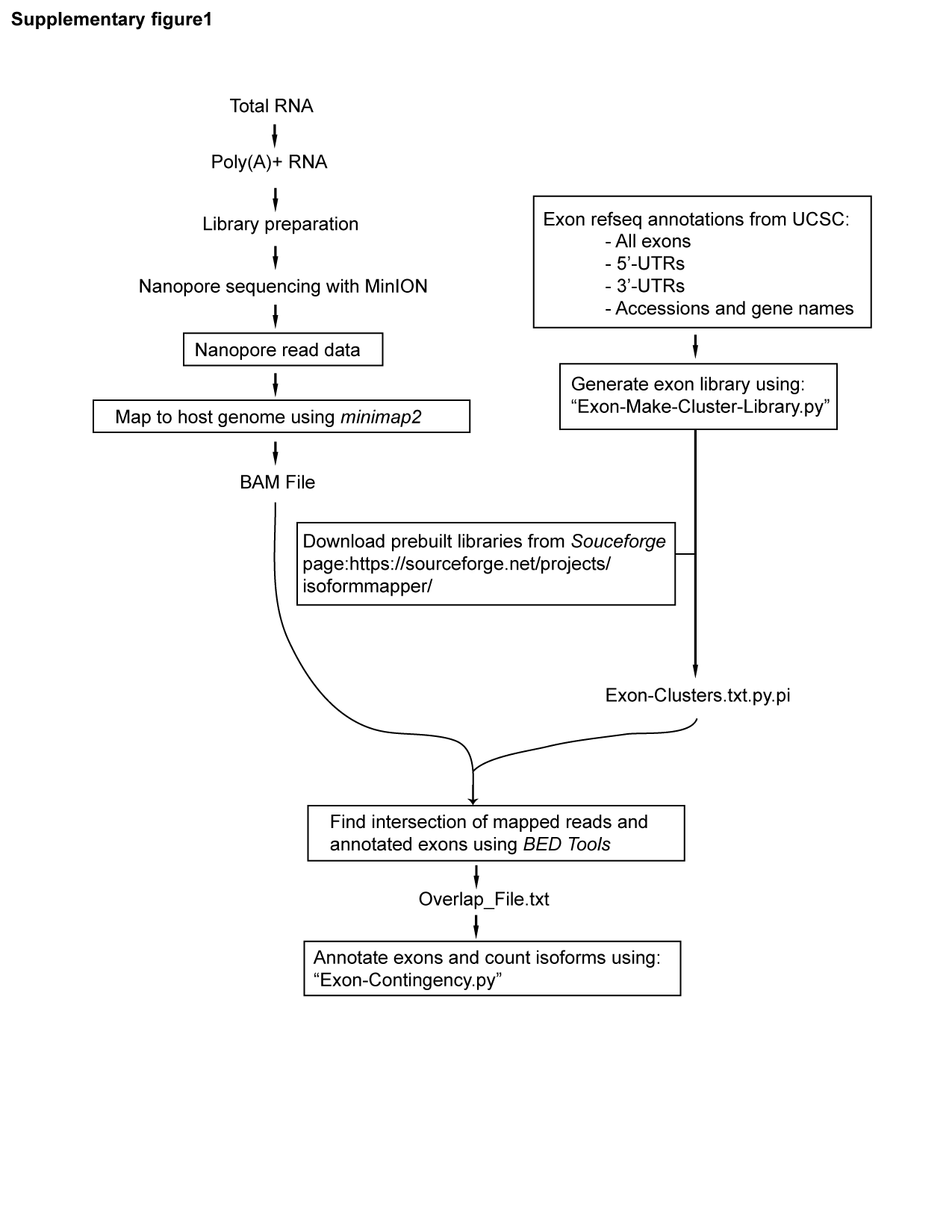

Supplement: Supplementary file 2 — Fig S1 [file JCMM-25-8352-s002.tif]
