# Supplementary material for: Nanopore sequencing reveals full‐length Tropomyosin 1 isoforms and their regulation by RNA‐binding proteins during rat heart development
Source: J Cell Mol Med. 2021 Jul 24;25(17):8352–62. doi: 10.1111/jcmm.16795 (PMC8419188; doi:10.1111/jcmm.16795)

Supplementary figure 2

Tpm2 transcripts in embryonic (E20) rat hearts

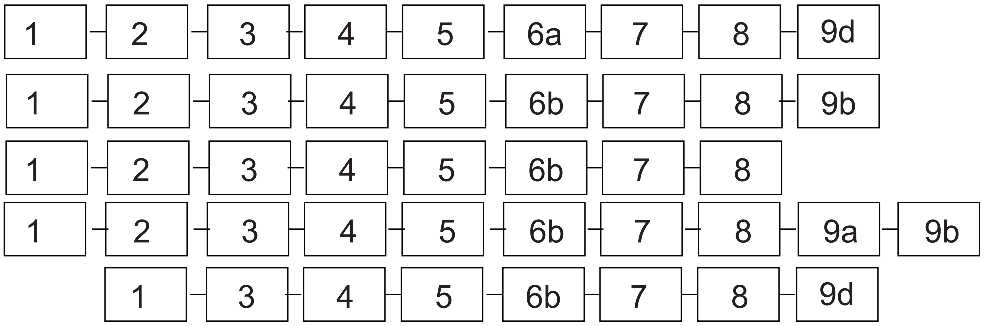

Tpm2 transcripts in adult (6M) rat hearts

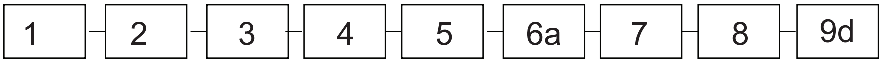

Tpm3 transcripts in embryonic (E20) rat hearts

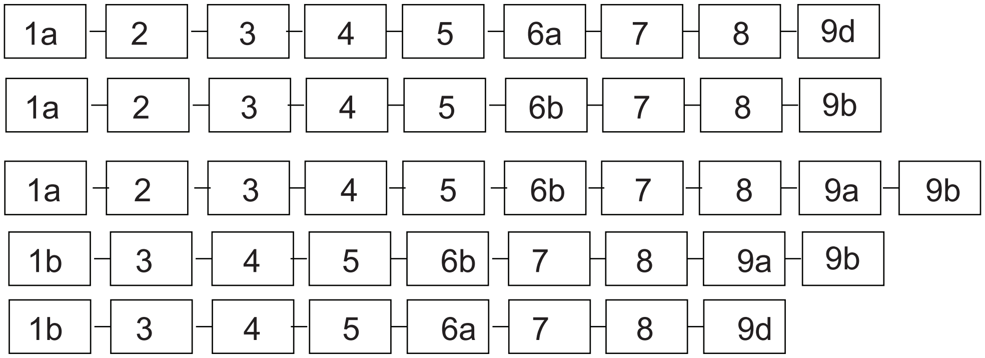

Tpm3 transcripts in adult (6M) rat hearts

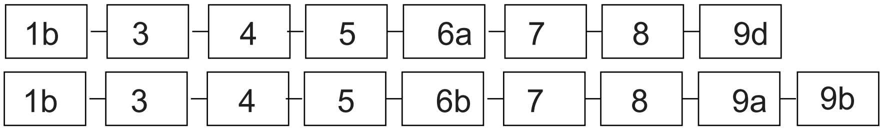

Tpm4 transcripts in embryonic (E20) rat hearts

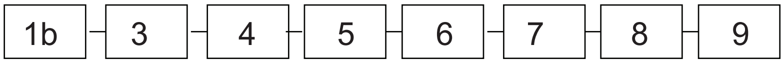

Tpm4 transcripts in adult (6M) rat hearts

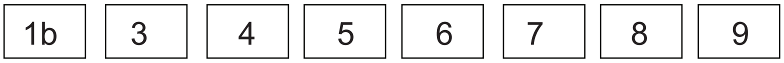

Supplement: Supplementary file 3 — Fig S2 [file JCMM-25-8352-s004.pdf]
