# Supplementary material for: Nanopore sequencing reveals full‐length Tropomyosin 1 isoforms and their regulation by RNA‐binding proteins during rat heart development
Source: J Cell Mol Med. 2021 Jul 24;25(17):8352–62. doi: 10.1111/jcmm.16795 (PMC8419188; doi:10.1111/jcmm.16795)
Supplement: Supplementary file 4 — Table S1 [file JCMM-25-8352-s001.docx]

**Supplemental Table 1**

| Gene name | Species | Gene ID | Analysis target | Forward primer (5’ to 3’) | Reverse primer (3’ to 5’) |
| --- | --- | --- | --- | --- | --- |
| *Tpm1* | Rat | ENSRNOG00000018184 | Short-*Tpm1* (Exon7- exon9b) | GAAGACAAATATGAAGAGGAGATCAAG | AATAGCAAACAGGAATAGCGTACA |
| *Tpm1* | Rat | ENSRNOG00000018184 | Long-*Tpm1*  (Exon7-exon9d) | GAAGACAAATATGAAGAGGAGATCAAG | CTGGTGCATACTAAGGTTTTCTTCTTT |
| *Tpm1* | Rat | ENSRNOG00000018184 | Exon4-exon6a | AAGATGCTGACCGAAAGTATGAAG | GCTTTCAAGGTCTGATCCATTATTCT |
| *Tpm1* | Rat | ENSRNOG00000018184 | Exon4-exon6b | AAGATGCTGACCGAAAGTATGAAG | TGAGCCTCCAGTGACTTCAA |
| *Hprt* | Rat | ENSRNOT00000045153.3 | Exon2-exon3 | CTCATGGACTGATTATGGACAGGAC | GCAGGTCAGCAAAGAACTTATAGCC |

**Table S1.** Primer list for PCR reactions.
